# Does dietary intake change during an intervention to reduce sedentary behavior and cardiovascular disease risk? A randomized comparative effectiveness trial

**DOI:** 10.1186/s40795-018-0223-1

**Published:** 2018-04-02

**Authors:** Kelsey Casey, Emily L. Mailey, Richard R. Rosenkranz, Aaron Swank, Elizabeth Ablah, Sara K. Rosenkranz

**Affiliations:** 1 Department of Human Nutrition, Kansas State University, 1105 Sunset Ave, 3rd Floor, Manhattan, KS 66502 USA; 2 Department of Kinesiology, Kansas State University, 8D Natatorium, Manhattan, KS 66502 USA; 3 Department of Preventive Medicine and Public Health, University of Kansas School of Medicine, 1010 North Kansas, Wichita, KS 672114 USA

**Keywords:** Sedentary, Insufficiently active women, Alternative healthy eating index, Dietary change, Dietary quality, Cardiovascular disease risk, Caloric compensation, Lifestyle factors changing together

## Abstract

**Background:**

Evidence from physical activity interventions suggests that women, in particular, may overcompensate for exercise energy expenditure by increasing caloric intake. Sedentary behavior and poor dietary quality are independent risk factors for many major chronic diseases, including cardiovascular disease (CVD). The primary purpose of this study was to determine whether insufficiently active women, accumulating less than 60 min per week of moderate-to-vigorous physical activity, alter caloric intake or dietary quality when participating in an 8-week intervention to reduce sedentary behavior and CVD risk. A secondary aim was to determine whether the two treatment groups differed from one another in dietary intake while participating in the intervention.

**Methods:**

Insufficiently active women (*n* = 49) working full-time sedentary jobs were randomized to one of two treatment groups to reduce sedentary behavior during the workweek: short-break (1–2 min breaks from sitting every half hour, SB), or long-break (15 min breaks from sitting twice daily, LB). Three-day food records were collected at baseline, week 4 and week 8. Dietary quality was assessed using the Alternative Healthy Eating Index 2010 (AHEI-2010). Risk factors for CVD were assessed at baseline and week 8.

**Results:**

For all participants, average caloric intake decreased significantly from baseline to week 8 by approximately 12% (∆ = − 216.0 kcals, *p* = 0.003). Average caloric intake decreased significantly over time for the SB group (∆ = − 369.6 kcals, *p* = 0.004), but not the LB group (∆ = − 179.5 kcals, *p* = 0.17). There was no significant difference between SB and LB groups with regard to calories from baseline to week 8 (F = 0.51, *p* = 0.48). Total AHEI-2010 scores did not decrease significantly for all participants (∆ = − 4.0, *p* = 0.14), SB (∆ = − 5.2, *p* = 0.16), or LB groups (∆ = − 4.5, *p* = 0.67).

**Conclusions:**

Following an 8-week intervention to reduce sedentary time, insufficiently active women decreased caloric intake over time, however there were no differences between SB and LB groups. In all participants, dietary quality was not altered over time. Future studies should explore sedentary reduction interventions compared to physical activity interventions as a means to create negative energy balance, as frequent sedentary breaks may be effective for improving health outcomes in women.

**Trial registration:**

ClinicalTrials.gov registration number NCT02609438, retrospectively registered November 20, 2015.

**Electronic supplementary material:**

The online version of this article (10.1186/s40795-018-0223-1) contains supplementary material, which is available to authorized users.

## Background

Excessive sedentary time is a contributing factor to many major chronic diseases, including cardiovascular disease (CVD) [1, 2], which is the leading cause of death among both males and females in the United States [3]. Recent studies suggest that time spent engaging in sedentary behavior impacts health, regardless of physical activity practices [4]. Despite this emerging evidence, sedentary behavior is becoming more common in the workplace [5]. Over 80% of adults now have sedentary jobs and 70–80% of a typical workday is spent sitting, usually in bouts of 20 min or more [5, 6].

Along with the adverse health impacts associated with sedentary behavior, prolonged sitting may provide the opportunity to consume food [7]. Eating food while watching TV, for example, may be distracting and inhibit the body’s ability to signal satiety [8], and has been associated with lower intake of fruits and vegetables along with higher intake of junk food [9, 10]. When comparing sedentary individuals to their more active counterparts, more active individuals tend to consume more healthful foods [11–13].

Cardiovascular disease is strongly affected by diet [14], yet the vast majority of the United States population fails to meet federal dietary recommendations [15]. Consumption of an overall diet that includes more healthy foods and lacks less healthy foods is more likely to have an impact on disease outcome than any one dietary component alone. The Alternate Healthy Eating Index 2010 (AHEI-2010) is an 11-component dietary index that characterizes an overall healthy diet. Higher scores on the AHEI-2010 have been shown to be associated with lower risk of chronic disease, particularly metabolic disease [15].

While diet and sedentary behaviors are both important considerations in a lifestyle intervention, lifestyle factors often change together. In general, as people attempt to change behavior to either achieve weight loss or health improvement benefits, they tend to alter more than one lifestyle behavior at a time [16]. Previous weight loss interventions targeting physical activity in both men and women indicate that some individuals may be more prone to compensation (indicated by failure to produce predicted weight loss) than others [17]. Women, in particular, who participate in weight loss studies tend to have lower success rates than their male counterparts, possibly due to caloric overcompensation [18, 19].

To our knowledge, there are currently no studies examining changes in dietary intake concurrent with an intervention to reduce sedentary time. The primary aim of the current study was to determine whether insufficiently active women would make incidental dietary changes while participating in an intervention aimed at reducing workplace sedentary time. More specifically, would participants exhibit changes in caloric intake or dietary quality? Due to a potential focus on health behavior changes overall, we hypothesized participants would decrease caloric intake and improve dietary quality while participating in the intervention. Our previously published primary findings from this study showed that there were differences between treatment groups (SB vs LB) for adherence as well as CVD risk factors [20]. Given these differences, a secondary aim was to determine whether the two treatment groups differed from one another for dietary intake while participating in the intervention.

## Methods

### Experimental design

The current study was part of a larger intervention to reduce sedentary time [20], which is outlined briefly below. Recruitment of participants began in March 2014 and the study was completed in June 2015. Participants were recruited through university email lists and flyers that were distributed at local businesses, and were then screened through an online survey that determined eligibility to participate in the study. Within the survey, participants were asked to describe their exercise habits during the past month to determine moderate-to-vigorous physical activity (MVPA). Once eligibility was established, packets were mailed to participants containing an informed consent document, accelerometer, accelerometer log, three-day food record, and instructions for wearing the accelerometer and completing the food record. After wearing the accelerometer for seven days, participants came to the lab for their baseline visit following a 10–12 h fast. Prior to the baseline appointment, participants had been randomized into one of two groups, the short-break (SB) group or the long-break (LB) group, at a 1:1 allocation ratio by an investigator not involved with testing, using a random digit generator in Microsoft Excel. At baseline, data were collected regarding participants’ anthropometrics and CVD risk factors. Anthropometric measurements included height, weight, and waist circumference. Cardiovascular disease risk factor assessments included total cholesterol, high-density lipoprotein (HDL), low-density lipoprotein (LDL), triglycerides, blood pressure (BP), and fasting glucose. Dietary intake was collected through a three-day food record, and physical activity was measured objectively through accelerometry. Participants were blinded to treatment group assignment until baseline data collection was complete, they then attended an individual 30-min orientation with a trained research assistant, during which their group assignment was explained, a planning worksheet was completed, and a list of computer/mobile applications that prompt activity breaks was provided. The SB group was instructed to take a break from sedentary behavior for one to two minutes every half hour throughout their workday. The LB group was instructed to take a break from sedentary behavior for 15 min, twice during their workday. Participants completed sedentary behavior logs daily that were submitted to researchers at the end of each week. To promote general adherence to the intervention, participants received a weekly email containing information and tips to reduce their sedentary time at work, and during the fourth week of the intervention participants received a phone call from a research assistant to discuss any questions or concerns. Accelerometry and dietary assessments were repeated at week 4 and week 8. All other assessments completed at baseline were repeated at week 8.

### Participants

**Fig. 1:**
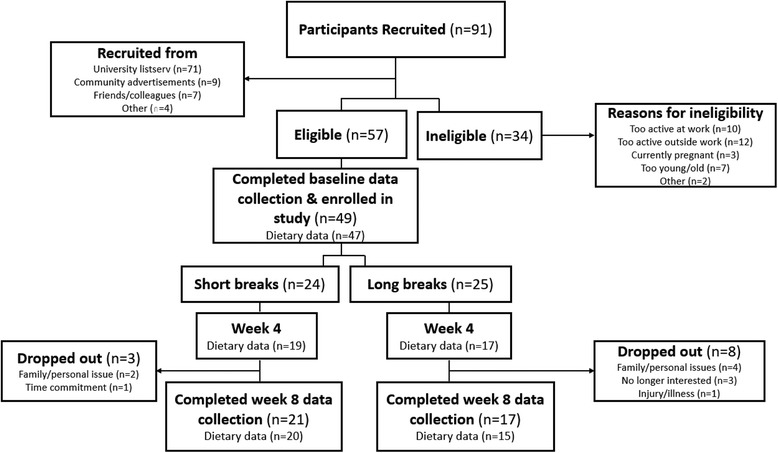
Recruitment and Retention of Participants

Figure 1 shows the flow diagram for recruitment and retention of participants in the current study. Of the 91 participants originally recruited, 49 were enrolled in the study (SB *n* = 24, LB *n* = 25), and 38 completed the study (SB *n* = 21, LB *n* = 17). Baseline characteristics (anthropometrics, CVD risk factors, and dietary intake) are shown in Table 1.

**Table 1:** Baseline Characteristics of SB and LB Groups

	SB Group (*n* = 24)		LB Group (*n* = 25)	
	Mean ± SD	Range	Mean ± SD	Range
Age (yr)	38.5 ± 8.7	22.0–50.0	39.9 ± 7.9	24.0–53.0
Weight (kg)	85.8 ± 25.5	48.2–167.6	86.1 ± 27.5	50.3–142.7
BMI (kg/m^2^)	32.4 ± 10.1^a^	18.9–65.9	32.4 ± 9.3^a^	20.3–50.5
Waist Circumference (cm)	102.4 ± 17.9	75.3–146.5	101.2 ± 21.9	75.2–136.8
Systolic BP (mmHg)	112.4 ± 11.9	93.3–134.5	113.6 ± 12.9	89.7–141.0
Diastolic BP (mmHg)	74.2 ± 8.5	62.7–89.5	74.7 ± 10.5	51.0–93.0
Triglycerides (mg/dL)	127.5 ± 54.6	45.0–219.0	153.9 ± 78.2	64.0–313.0
Total cholesterol (mg/dL)	172.6 ± 28.8	120.0–223.0	190.9 ± 24.8	145.0–234.0
HDL (mg/dL)	51.3 ± 15.4	24.0–76.0	48.7 ± 16.3	15.0–80.0
LDL (mg/dL)	94.9 ± 26.0	42.0–147.0	109.6 ± 24.1	62.0–150.0
Glucose (mg/dL)	98.0 ± 14.4	81.0–135.0	100.8 ± 32.8^a^	72.0–243.0
Caloric Intake (per day)	1944 ± 539	873–3034	1725 ± 386	999–2543
% Carbohydrate	44.5 ± 7.6	30.5–58.1	44.9 ± 8.9	24.6–66.6
% Protein	16.8 ± 4.2	10.8–28.2	17.3 ± 4.3	10.7–29.9
% Fat	37.1 ± 6.6	21.7–46.4	36.8 ± 6.	22.7–45.9
AHEI-2010	52.4 ± 16.3	29.5–90.5	54.5 ± 12.0	29.5–76.0

*SB* Short-break, *LB* Long-break, *BMI* Body mass index, *Systolic BP* Systolic blood pressure, *Diastolic BP* Diastolic blood pressure**,** *HDL* High density lipoprotein, *LDL* Low density lipoprotein; *% Carbohydrates* Percentage of total calories from carbohydrate, *% Protein* Percentage of total calories from protein, *% Fat* Percentage of total calories from fat

^a^Elevated CVD risk

Participants were 49 healthy women (aged 25–50 years) who worked 35 or more hours per week in a job where at least 80% of the workday was spent seated. All participants were: insufficiently active, accumulating less than 60 min per week of MVPA [21]; not pregnant; and not currently trying to change physical activity or dietary behaviors. Written informed consent was obtained from all those who participated. The experimental protocol was approved by the Institutional Review Board at Kansas State University in Manhattan, KS (IRB #7031) and conforms to the Declaration of Helsinki.

### Tests and measurements

The detailed procedures and methods have been previously published [20]. Briefly, at the baseline and 8-week assessment periods, height, weight, and waist circumference were measured in light clothing with shoes removed. Body mass index (BMI) was calculated as weight (kg) divided by height (m) squared. Blood pressure was measured using an automated BP cuff according to standard procedures reported previously. A fasting whole-blood sample was taken using a finger puncture to measure total cholesterol, HDL cholesterol, LDL cholesterol, triglycerides, and glucose. All measurements were taken by a trained research assistant.

Dietary intake was measured through three-day food records at each of the three time points. Participants recorded their diet on two weekdays and one weekend day, which is a dietary assessment method that has been previously validated [22]. The participants were given written instruction on how to complete the log, and reviewed their logs with a trained research assistant at each assessment to clarify any unclear entries. Dietary intake was analyzed using Nutritionist Pro nutrition analysis software (version 6.1.0, Axxya Systems, Stafford, TX) by a trained research assistant.

Dietary quality was assessed using the AHEI-2010. Additional file 1 shows the scoring method for the components of the AHEI-2010. Each component receives a score of 0–10 based proportionally on the intake as compared to guidelines, with a possible total score of 110. Higher scores are given for higher consumption of fruits, vegetables, whole grains, nuts, ω-3 fatty acids, and polyunsaturated fatty acids (PUFAs). Conversely, lower scores are given for higher consumption of sugar-sweetened beverages (SSBs) and fruit juices, red and/or processed meat, trans fats, and sodium. The alcohol score is based on moderate drinking, with a maximum score given for moderate alcohol intake (0.5–1.5 drinks/day), the lowest score given to those who drank in excess (> 1.5 drinks/day), and a score of 2.5 given to non-drinkers [15]. Scores were determined using data retrieved from three-day food records, reports generated from Nutritionist Pro, and additionally, individual food composition data automatically generated from Nutritionist Pro. Data collected for all components were averaged over three days and used the guidelines below for scoring. Additional file 2 shows locations of AHEI-2010 components derived using Nutritionist Pro. Additional information regarding AHEI-2010 scoring can be found in Additional file 3 [23–25].

As part of the larger intervention to reduce sedentary time [20], physical activity was measured objectively by using Actigraph (Pensacola, FL) GT3X physical activity monitors at each of the three time points. Accelerometers were worn at the waist over the right hip for seven days at each assessment period. Data were collected over 10-s epochs at a sampling frequency of 30 Hz across three axes, and were downloaded and analyzed using ActiLife 6.0. Sedentary behavior was defined as periods when counts per minute were ≤ 100 [26], light activity ranges were 100–1951, and moderate activity ranges were 1952–5724 [27]. Since this intervention targeted sitting time during the workday, a time filter was used to restrict the data to participants’ working hours. A valid day was considered to be any day that the accelerometer was worn for at least 10 h. Participants also completed daily activity logs throughout the 8-week intervention.

Adherence to intervention condition was measured using the participants’ activity logs. For the SB group, full adherence was defined as taking 12 or more breaks during the workday. Days when SB participants took 6 breaks or fewer, or did not submit a log, were coded as non-adherence. For the LB group, full adherence was defined as taking 2 or more breaks totaling 25 min or more during the workday. Any days that LB participants did not report an activity break longer than 10 min or did not submit an activity log were considered days of non-adherence.

### Statistical analyses

As part of the overall study design, a preliminary power analysis (80% power, *a* = 0.05) estimated that a sample size of 28 per group would be necessary in order to detect a medium-size reduction (*d* = 0.50) in sedentary time at work from baseline to week 8 for the entire cohort. This study was not powered to detect changes in the dietary intake or CVD risk factors. Detailed information on power analyses have been previously published [20]. Statistical analyses were performed using SPSS Statistics for Windows, Version 22.0 (Armonk, NY: IBM Corp). Parametric assumptions were tested for all independent and dependent variables, and if assumptions were not met, data were transformed (Lg10). Two-way repeated measures analysis of variance (ANOVA) was used to determine changes between and within groups across the assessment periods regarding dietary intake variables. In order to test for changes in CVD risk factors, independent t-tests were used to determine differences between SB and LB groups, and paired t-tests were used to assess differences between time points (baseline and week 8). Correlations between dietary factors, and adherence were tested using Pearson product-moment correlations. For data that did not meet parametric assumptions after transformation (glucose, vitamin K, monounsaturated fatty acid, total fat %, vitamin D, saturated fat, trans fat AHEI-2010 score, and alcohol AHEI-2010 score), Friedman’s tests were used to determine within-and between-group differences, Wilcoxon signed-rank tests were used to determine changes in dietary intake and CVD risk over time, and Spearman correlation to test correlations. Data are shown as the mean ± standard deviation. For all tests, statistical significance was set at *p* < 0.05. Where multiple tests were performed, Bonferroni corrections were used to account for the increased chance of type I error.

## Results

Considering all participants, average caloric intake (baseline: 1836.7 ± 478.2 kcals, week 4: 1732.6 ± 534.4 kcals, week 8: 1620.7 ± 470.0 kcals) decreased significantly from baseline to week 8 (*F* = 10.51, *p* = 0.003), with no difference between SB and LB groups (*F* = 0.51, *p* = 0.48), and no significant group x time interaction (*F* = 0.75, *p* = 0.40).

**Fig. 2:**
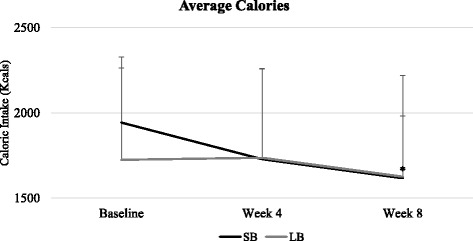
Caloric Intake over Three Assessment Periods for SB and LB Groups. Short-break (SB) group shown in black. Long-break (LB) group shown in gray. Error bars indicate SD. Baseline (SB *n* = 24; LB *n* = 23), week 4 (SB *n* = 19; LB *n* = 17), week 8 (SB *n* = 20, LB *n* = 15). * Statistically significant decrease from baseline to week 8 for SB group (*p* = 0.004)

Figure 2 shows average caloric intake (mean ± SD) for both SB and LB groups at each time point. Average caloric intake decreased significantly in the SB group over time (baseline: 1986.4 ± 542.2 kcals, week 4: 1728.8 ± 541.5 kcals, week 8: 1616.8 ± 355.9 kcals; *F* = 11.38, *p* = 0.004). For the LB group, average caloric intake (baseline: 1805.4 ± 412.5 kcals, week 4: 1717.9 ± 541.5 kcals, week 8: 1625.9 ± 603.8 kcals) did not change significantly over time (*F* = 2.09, *p* = 0.17).

Additional file 4 shows mean AHEI-2010 scores for participants who completed food records at all three time points. There were no significant changes in total AHEI-2010 scores over time (*F* = 1.94, *p* = 0.17), no differences between SB and LB groups (*F* = 0.01, *p* = 0.94), and no significant group x time interaction (*F* = 0.94, *p* = 0.34). There were no significant changes in any of the individual AHEI-2010 component scores from baseline to week 8 for all participants (*p* > 0.05).

Table 2 shows the mean of the macronutrient distribution, which is the percent of total calories from protein, fat, and carbohydrates, at all three time points. There were no significant changes in macronutrient distribution for all participants over time, no differences between SB and LB groups (protein: *F* = 0.30, *p* = 0.59; fat: *F* = 0.14, *p* = 0.71; carbohydrate: *F* = 1.12, *p* = 0.30), and no significant group x time interaction (protein: *F* = 0.06, *p* = 0.81; fat: *F* = 0.06, *p* = 0.81; carbohydrate: *F* = 0.00, *p* = 0.99).

**Table 2:** Average Macronutrient Distribution During Three Assessment Periods for SB, LB, and All Participants

		Baseline	Week 4	Week 8	*F*	*p*-value
Protein (%)	SB	16.8 ± 4.2	16.9 ± 4.4	17.4 ± 4.6		
	LB	17.3 ± 4.3	15.2 ± 3.8	16.5 ± 3.6		
	All	17.1 ± 4.2	16.1 ± 4.2	17.0 ± 4.2	0.53	0.47
Fat (%)	SB	37.1 ± 6.6	35.9 ± 10.6	38.1 ± 3.9		
	LB	36.8 ± 6.8	33.4 ± 7.6	37.2 ± 8.0		
	All	37.0 ± 6.7	34.7 ± 9.3	37.7 ± 5.9	2.68	0.11
Carbohydrate (%)	SB	44.5 ± 7.6	43.8 ± 8.2	43.8 ± 5.5		
	LB	44.9 ± 8.9	50.2 ± 8.5	45.7 ± 9.4		
	All	44.7 ± 8.2	46.8 ± 8.8	44.6 ± 7.3	3.87	0.06

*SB* Short-break, *LB* Long-break, *All* All participants

Baseline (SB *n* = 24; LB *n* = 23; All *n* = 47), week 4 (SB *n* = 19; LB *n* = 17; All *n* = 36), week 8 (SB *n* = 20, LB *n* = 15; All = 35)

As previously reported [20], average minutes of sedentary time per workday decreased significantly in the SB group from baseline to week 8 (baseline: 433.4 ± 45.5 min, week 8: 397.8 ± 49.5 min, *p* = 0.03), but did not significantly change in the LB group (baseline: 415.7 ± 45.8 min, week 8: 421.1 ± 40.3 min, *p* = 0.68). In all participants, there were no significant correlations between changes in sedentary time and changes in caloric intake (*r* = − 0.12, *p* = 0.51), total AHEI-2010 scores (*r* = 0.20, *p* = 0.27), AHEI-2010 components (*p* > 0.05), or macronutrient distribution (protein: *r* = − 0.08, *p* = 0.64; fat: *r* = − 0.04, *p* = 0.81; carbohydrate: *r* = − 0.13, *p* = 0.47).

Cardiovascular disease risk factors at baseline are shown in Table 1. Cut-points for CVD risk factors were determined using the American College of Sports Medicine guidelines [28]. These risk factors include: BP ≥140/90 mmHg, LDL ≥130 mg/dL, HDL < 40 mg/dL, BMI ≥30 kg/m^2^, or fasting blood glucose ≥100 mg/dL. Accumulating less than 30 min of physical activity three days per week is also a risk factor. The presence of two or more of these risk factors increases overall risk for CVD. Of the 49 participants, 34 had two or more risk factors (SB: *n* = 15, LB: *n* = 19).

There were no statistically significant changes for CVD risk factors when examining all participants together. The SB group had a significant reduction in fasting blood glucose from baseline to week 8 (baseline: 98.8 ± 15.2 mg/dL, week 8: 94.6 ± 13.8 mg/dL, *p* = 0.009), while the LB group showed no significant change in any of the CVD risk factors.

Thirty-two percent of participants randomized into the LB group dropped out by the end of the study, whereas only 13% of SB group participants dropped out. We also examined whether or not adherence to the intervention was associated with dietary quality, caloric intake, or CVD outcomes. For SB group participants, percentage of full adherence was negatively correlated with percent change in caloric intake with a moderate effect size (*r* = 0.47), such that participants who had more days of full adherence to the intervention protocol exhibited greater reductions in caloric intake. There were no associations between adherence and dietary quality or CVD outcomes. For LB group participants, there were no correlations between adherence and dietary quality, caloric intake, or CVD outcomes.

## Discussion

With regard to overall caloric intake, there is much more known about diet and physical activity interventions than diet and sedentary behavior interventions. Previous research on dietary changes during physical activity interventions suggests that women may overcompensate for increased energy expenditure by increasing caloric intake [18, 19]. To our knowledge, no studies have examined incidental changes in caloric intake when participating in a study intended to reduce sedentary time. In the current study, participants in the LB group did not change caloric intake significantly, but participants in the SB group reduced their caloric intake, compared to baseline, ~ 215 cal at week 4, and ~ 330 cal at week 8. Since sedentary time has been shown to be associated with increased opportunities to snack, it is possible that an overall reduction in caloric intake was seen due to disrupted opportunities to consume food during sedentary time. Due to the nature of the LB group intervention, given that previous research has suggested that women tend to compensate calorically when increasing physical activity, it is possible that the women in the LB group partially compensated for what they might have viewed as increased physical activity [18, 19].

There is currently limited information available about the diet quality of sedentary individuals. Typically, research examining this question categorizes those who do not meet physical activity guidelines as sedentary. Individuals not meeting physical activity guidelines regularly have higher intake of saturated fats, dietary cholesterol, and lower intake of fruits and vegetables as compared to those engaging in at least 150 min of moderate activity or 60 min of vigorous activity per week [12]. The Sedentary Behaviour Research Network (SBRN) defines sedentary behavior as any waking behavior characterized by an energy expenditure ≤1.5 metabolic equivalents of tasks (METs) while in a seated or reclining posture, and not just absence of MVPA [29]. To our knowledge, there are no studies available on dietary quality and sedentary behavior that use this definition to categorize participants as sedentary. The current study included participants who were not meeting physical activity guidelines, but who also were employed in full time sedentary jobs, based on the SBRN definition.

Since our participants were asked to decrease sedentary behavior rather than increase physical activity, it was unknown whether dietary quality would increase when sedentary behavior was targeted. Results indicated that there were no significant changes in overall dietary quality between or within groups throughout the study. Total AHEI-2010 scores for all participants at baseline (53.4 ± 14.2) were above the national average for women of 39.0 [15], and remained above the national average at week 8 (48.4 ± 12.5). It is important to recognize that previous research has shown that caloric intake and AHEI-2010 scores are associated [30] and that with reductions in caloric intake, scores might decrease, as consuming fewer calories allows for fewer opportunities to consume nutrient-dense foods. Although participants did not increase their dietary quality, the SB group was able to maintain their dietary quality, while also significantly decreasing caloric intake, and the LB group did not significantly change dietary quality or caloric intake. Even though participants in the SB group decreased their caloric intake without significantly negatively impacting their AHEI-2010 scores, there are other dietary concerns to keep in mind.

Macronutrient distribution remained unchanged throughout the study. The acceptable macronutrient distribution range (AMDR) is 10–35% of total calories from protein, 20–35% of total calories from fat, and 45–65% of total calories from carbohydrates [31]. All participants were consuming higher than recommended total calories from fat, and total fat was associated with higher LDL cholesterol, which is a risk factor for CVD. Participants also consumed lower than recommended total calories from carbohydrates. Despite the caloric intake reduction in the SB group, macronutrient distributions did not shift toward the AMDR ranges, perhaps factoring in to the lack of CVD risk factor changes overall.

## Experimental considerations

### Strengths of current study

There are several strengths of the current study, which add to the current body of literature. To our knowledge, this is the first study to examine the relationship between an intervention aimed at reducing sedentary time in the workplace and incidental dietary changes. Previous studies examining multiple lifestyle factors changing together have focused on dietary changes with physical activity or weight loss interventions. In contrast, our participants were not asked to increase physical activity, but to decrease sedentary time during their workday. Acute dietary intake was collected at the time of consumption for three days, and participants were not asked to recall their diet, so there is less chance of recall bias. Further, diet was evaluated, not only regarding calories and macronutrients, but also using an overall index of dietary quality.

### Limitations of current study

There were some limitations in this study that should be considered when interpreting findings. Rather than in-person training for completion of the food records, participants received the food record with written instructions via mail. A trained research assistant did, however, review the record with the participants for any unclear entries when they came in for assessments. With food records, there are also potential biases to consider as participants may change their eating patterns when they know they are being evaluated. However, this potential bias would apply to both the SB and LB groups equally. The study design required the participants to be actively engaged in the study every workday for eight weeks, which may have caused a large participant burden. Additionally, the quality of completion as well as the number of food records submitted throughout the study may have dropped due to participant fatigue. Data collection for some participants occurred during the winter holiday season. Eating habits as well as ability/willingness to comply with the intervention may have been affected by travel, days off from work, and other demands during this time. Again, this limitation would have applied to both SB and LB participants. Lastly, the limited sample size of the study may reduce the generalizability of results.

### Future directions

Future research should compare dietary changes in physical activity interventions to dietary changes in sedentary reduction interventions to determine whether women are less prone to caloric compensation in sedentary reduction interventions. Additionally, future interventions should target both decreases in sedentary behavior and improvements in dietary quality. Given that women in the current study did not seem to compensate for participating in the intervention with increased incidental calories, targeting dietary quality in the intervention might result in improvements in sedentary behavior and improved dietary quality along with caloric reductions, perhaps improving CVD risk factors further.

## Conclusions

Our findings add important information to the existing body of literature investigating lifestyle changes occurring together. In women not meeting physical activity guidelines and working in sedentary occupations, an intervention targeting sedentary breaks led to incidental decreases in caloric intake without decrements in dietary quality.

## Additional files

Additional file 1: Scoring of AHEI-2010 Components. A brief overview of the scoring methods for the AHEI-2010, including maximum and minimum scores for each component. (DOCX 12 kb)

Additional file 2: Nutritionist Pro Reports for AHEI-2010 Scoring**.** A summary of the Nutritionist Pro reports that were used for scoring each AHEI-2010 component. (DOCX 12 kb)

Additional file 3: AHEI-2010 Scoring Method. A detailed description of how to use the various reports within Nutritionist Pro to score each component of the AHEI-2010. (DOCX 13 kb)

Additional file 4: Average AHEI-2010 Scores During Three Assessment Periods for SB, LB, and All Participants. Mean scores for each AHEI-2010 component at baseline, week 4, and week 8. (DOCX 15 kb)

## Abbreviations

AHEI: Alternate Healthy Eating Index
AMDR: Acceptable macronutrient distribution range
BMI: Body mass index
BP: Blood pressure
CVD: Cardiovascular disease
HDL: High-density lipoprotein
LB: Long-break
LDL: Low-density lipoprotein
MVPA: Moderate-to-vigorous physical activity
PUFA: Polyunsaturated fatty acid
SB: Short-break
SBRN: Sedentary Behaviour Research Network
SSBs: Sugar-sweetened beverages
