# Supplementary material for: Does dietary intake change during an intervention to reduce sedentary behavior and cardiovascular disease risk? A randomized comparative effectiveness trial
Source: BMC Nutr. 2018 Apr 2;4:16. doi: 10.1186/s40795-018-0223-1 (PMC7050876; doi:10.1186/s40795-018-0223-1)
Supplement: Supplementary file 1 — Scoring of AHEI-2010 Components. A brief overview of the scoring methods for the AHEI-2010, including maximum and minimum scores for each component. (DOCX 12 kb) [file 40795_2018_223_MOESM1_ESM.docx]

| Component | Maximum Score (10) | Minimum Score (0) |
| --- | --- | --- |
| Whole Fruit | ≥4 servings/day | 0 servings/day |
| Total Vegetables | ≥2.5 cups/day | 0 cups/day |
| Whole Grains | 75 g/day | 0 g/day |
| SSBs and Fruit Juice | 0 oz/day | ≥8 oz/day |
| Nuts and Legumes | ≥1 oz/day | 0 oz/day |
| Red and/or Processed Meat | 0 servings/day | ≥1.5 servings/day |
| Trans Fat | ≤0.5% of energy/day | ≥4% of energy/day |
| ω-3 Fats | 250 mg/day | 0 mg/day |
| PUFAs | ≥10% of energy/day | ≤2% of energy/day |
| Alcohol | 0.5-1.5 drinks/day | ≥2.5 drinks/day |
| Sodium | Lowest decile | Highest decile |

**Additional file 1
Scoring of AHEI-2010 Components**
