# Supplementary material for: Does dietary intake change during an intervention to reduce sedentary behavior and cardiovascular disease risk? A randomized comparative effectiveness trial
Source: BMC Nutr. 2018 Apr 2;4:16. doi: 10.1186/s40795-018-0223-1 (PMC7050876; doi:10.1186/s40795-018-0223-1)
Supplement: Supplementary file 2 — Nutritionist Pro Reports for AHEI-2010 Scoring. A summary of the Nutritionist Pro reports that were used for scoring each AHEI-2010 component. (DOCX 12 kb) [file 40795_2018_223_MOESM2_ESM.docx]

Additional file 2
Nutritionist Pro Reports for AHEI-2010 Scoring

| Component | Nutrition Summary Report | MyPlate  Summary Report | Food Analysis | Food Record |
| --- | --- | --- | --- | --- |
| Whole Fruit |  | **X** |  |  |
| Total Vegetables |  | **X** |  |  |
| Whole Grains |  |  | **X** | **X** |
| SSBs and Fruit Juice |  |  |  | **X** |
| Nuts and Legumes |  |  |  | **X** |
| Red and/or Processed Meat |  |  |  | **X** |
| Trans Fat | **X** |  |  |  |
| ω-3 Fats | **X** |  |  |  |
| PUFAs | **X** |  |  |  |
| Alcohol | **X** |  |  | **X** |
| Sodium | **X** |  |  |  |
