# Supplementary material for: Does dietary intake change during an intervention to reduce sedentary behavior and cardiovascular disease risk? A randomized comparative effectiveness trial
Source: BMC Nutr. 2018 Apr 2;4:16. doi: 10.1186/s40795-018-0223-1 (PMC7050876; doi:10.1186/s40795-018-0223-1)
Supplement: Supplementary file 3 — AHEI-2010 Scoring Method. A detailed description of how to use the various reports within Nutritionist Pro to score each component of the AHEI-2010. (DOCX 13 kb) [file 40795_2018_223_MOESM3_ESM.docx]

**Additional file 3
AHEI-2010 Scoring Method**

The Nutrition Summary report was used to obtain average three-day macronutrient composition, total caloric intake, and data on the individual AHEI-2010 components: trans fats, PUFAs, ω-3 fatty acids, sodium, and alcohol intake. Research assistants calculated the percentage of overall calories from trans fats and PUFAs using the grams per day in order to score these two categories. The direct output in milligrams per day provided was used for scoring ω-3 fats.

Fruit and vegetable data were obtained through the MyPlate summary report. In this report, research assistants were able to view each individual food component consumed, that component’s MyPlate category, and the amount that was consumed. A serving of whole or dried fruit was equivalent to 0.5 cups, and fruits mixed with non-fruit foods were considered by half weighting. Vegetable intake was scored by cups/day of whole vegetables, excluding potatoes, starchy vegetables, vegetable juices and vegetable sauces. Half weight was given to mixed vegetable foods such as soups as well as vegetables with sauces.

Nuts and legumes, SSBs and fruit juices, and red and/or processed meat data were obtained from the three-day food records. Intakes of each component were averaged over the three-day period at each time point. Nuts, legumes, and seeds along with nut butters and tofu were all considered for the nut and legume category. Half weight was assigned for nut butter sandwiches, soy milk, tofu soups, and mixed dishes containing nuts/legumes. One serving of SSBs or fruit juices was defined as 8 fl oz. Red and/or processed meat included any beef, pork or processed meat, with 3.5 oz considered as one serving. Half weight was given to mixed foods such as sandwiches, broths, or dishes containing meat and vegetables.

Whole-grain consumption data were obtained through several steps. Foods in the three-day food record that met the AHEI-2010 guidelines such as brown rice and popcorn were recorded in grams. All other grain products were individually evaluated for their carbohydrate-to-fiber ratio. Grams of carbohydrates and grams of fiber were obtained through the analysis feature for each food item. Foods that had a carbohydrate-to-fiber ratio less than or equal to 10:1 were considered whole grains [31]. Serving size was determined based on the type of grain; 1 slice of bread, 0.5 cups pasta, and 1 cup of cereal were all considered one serving.

Overall sodium intake in milligrams was divided into deciles for each time point and these deciles were used for scoring. The lowest decile was given the highest score of 10, the highest decile was given the lowest score of 0, and all other deciles were given scores 1-9 accordingly. When the Nutrient Analysis report indicated that the participant had consumed alcohol, the research assistant used the three-day food records to calculate the per day average consumption, with serving sizes defined as 141.75 g of wine, 340.20 g of beer, or 42.53 g of liquor, and half weight assigned to cocktails [15].
