# Supplementary material for: Does dietary intake change during an intervention to reduce sedentary behavior and cardiovascular disease risk? A randomized comparative effectiveness trial
Source: BMC Nutr. 2018 Apr 2;4:16. doi: 10.1186/s40795-018-0223-1 (PMC7050876; doi:10.1186/s40795-018-0223-1)
Supplement: Supplementary file 4 — Average AHEI-2010 Scores During Three Assessment Periods for SB, LB, and All Participants. Mean scores for each AHEI-2010 component at baseline, week 4, and week 8. (DOCX 15 kb) [file 40795_2018_223_MOESM4_ESM.docx]

Additional file 4
Average AHEI-2010 Scores During Three Assessment Periods for SB, LB, and All Participants

|  | | | | | | | | | |
| --- | --- | --- | --- | --- | --- | --- | --- | --- | --- |
|  | SB Group | | | LB Group | | | All Participants | | |
|  | Baseline (n=24) | Week 4 (n=19) | Week 8 (n=20) | Baseline (n=23) | Week 4 (n=17) | Week 8 (n=15) | Baseline (n=47) | Week 4 (n=36) | Week 8 (n=35) |
| Whole Fruit | 3.0±3.0 | 1.7±2.0 | 2.3±2.3 | 2.6±2.4 | 3.4±3.2 | 3.5±2.9 | 2.8±2.7 | 2.5±2.8 | 2.8±2.6 |
| Total Vegetable | 3.2±2.2 | 4.0±2.6 | 4.1±2.8 | 3.4±2.3 | 2.5±1.8 | 3.0±1.9 | 3.3±2.2 | 3.3±2.4 | 3.6±2.5 |
| Whole Grains | 4.5±3.6 | 5.4±3.9 | 3.3±3.4 | 5.0±4.2 | 4.7±4.1 | 4.1±3.9 | 4.7±3.9 | 5.1±3.9 | 3.6±3.6 |
| SSBs and Fruit Juices | 5.4±4.4 | 6.4±4.6 | 6.1±4.2 | 6.4±4.4 | 4.3±4.2 | 6.2±4.4 | 5.9±4.3 | 5.4±4.5 | 6.1±4.2 |
| Nuts and Legumes | 5.3±4.3 | 6.1±4.4 | 4.4±4.4 | 4.6±4.4 | 4.9±4.1 | 4.7±4.8 | 4.9±4.3 | 5.5±4.3 | 4.5±4.5 |
| Red/ Processed Meat | 4.2±3.4 | 3.6±2.9 | 4.1±2.9 | 5.0±2.8 | 5.5±3.4 | 4.2±2.4 | 4.6±3.1 | 4.5±3.2 | 4.1±2.7 |
| Trans Fat | 9.9±0.3 | 9.3±1.1 | 9.3±1.3 | 9.7±0.7 | 9.5±0.9 | 9.1±2.6 | 9.8±0.5 | 9.4±1.0 | 9.2±1.9 |
| ω-3 Fats | 3.2±3.3 | 1.9±2.2 | 2.7±3.3 | 3.1±3.5 | 2.3 ± 3.2 | 2.1±2.6 | 3.1±3.3 | 2.1±2.7 | 2.4±3.0 |
| PUFA | 4.9±2.4 | 4.2±2.6 | 3.5±2.7 | 5.0±2.7 | 2.7±2.2 | 4.1±2.5 | 4.9±2.5 | 3.5±2.5 | 3.8±2.6 |
| Alcohol | 4.1±3.1 | 3.7±2.8 | 2.4±0.6 | 3.8±2.9 | 3.7 ± 3.1 | 3.5±2.6 | 3.9±3.0 | 3.7±2.9 | 2.9±1.8 |
| Sodium | 4.8±3.4 | 4.9±3.5 | 5.3±3.4 | 6.0±2.4 | 5.8 ±2.5 | 5.5±2.5 | 5.4±3.0 | 5.4±3.0 | 5.4±3.0 |
| Total Score | 52.4±16.3 | 51.2±14.8 | 47.2±13.1 | 54.5±12.0 | 49.3±11.0 | 50.0±11.8 | 53.4±14.2 | 50.3±13.0 | 48.4±12.5 |
| SB, short-break; LB, long-break Maximum possible score for individual components: 10, maximum total score possible: 110 There were no significant differences within or between groups at any time points for AHEI-2010 components (*p*>0.05). | | | | | | | | | |
